# High Pressure Air Jet in the Endoscopic Preparation Room: Risk of Noise Exposure on Occupational Health

**DOI:** 10.1155/2015/610582

**Published:** 2015-02-01

**Authors:** King-Wah Chiu, Lung-Sheng Lu, Cheng-Kun Wu

**Affiliations:** Division of Gastroenterology and Hepatology, Department of Internal Medicine, Kaohsiung Chang Gung Memorial Hospital and Chang Gung University College of Medicine, 123 Ta-Pei Road, Niao-Sung, Kaohsiung 833, Taiwan

## Abstract

After high-level disinfection of gastrointestinal endoscopes, they are hung to dry in order to prevent residual water droplets impact on patient health. To allow for quick drying and clinical reuse, some endoscopic units use a high pressure air jet (HPAJ) to remove the water droplets on the endoscopes. The purpose of this study was to evaluate the excessive noise exposure with the use of HPAJ in endoscopic preparation room and to investigate the risk to occupational health. Noise assessment was taken during 7 automatic endoscopic reprocessors (AERs) and combined with/without HPAJ use over an 8-hour time-weighted average (TWA). Analytical procedures of the NIOSH and the ISO for noise-induced hearing loss were estimated to develop analytic models. The peak of the noise spectrum of combined HPAJ and 7 AERs was significantly higher than that of the 7 AERs alone (108.3 ± 1.36 versus 69.3 ± 3.93 dBA, *P* < 0.0001). The risk of hearing loss (HL > 2.5 dB) was 2.15% at 90 dBA, 11.6% at 95 dBA, and 51.3% at 100 dBA. The odds ratio was 49.1 (95% CI: 11.9 to 203.6). The noise generated by the HPAJ to work over TWA seriously affected the occupational health and safety of those working in an endoscopic preparation room.

## 1. Introduction

According to the British Society of Gastroenterology guidelines for the decontamination of equipment used for gastrointestinal (GI) endoscopy, there are three important steps: cleaning by manual washing, disinfection in automatic endoscopic reprocessors (AERs), and drying in order to prevent residual water droplets impact on patient health [1]. For patient safety, regular maintenance of the AER [2], adequate manual washing of the working channel for longer small intestinal scopes and colon scopes in particular [3], scheduled high-level disinfection (HLD) with the AER [4], and overnight spraying of the AER with alcohol [5] are very important to prevent infections after GI endoscopy. In an academic teaching hospital, the large clinical loading of GI endoscopy may mean that the time required to dry the scopes overnight is not feasible. To increase the speed of clinical reuse and drying, a high pressure air jet (HPAJ) is used to remove residual water from the surface of endoscopes after HLD disinfection with AER in some GI endoscopic units in Taiwan. The concept is similar to an outdoor high pressure car wash and dry. However, it would seem that the noise resulting from using such a system would compromise the health and safety of the technicians. The Occupational Safety and Health Act of 1970 was passed to prevent workers from being killed or seriously harmed at work [6]. The law requires employers to provide their employees with working conditions that are free of known dangers. Clinically, noise assessment of dental procedures has been reported with regard to hospital occupational health [7]. The Occupational Safety and Health Administration (OSHA) was established after the act was enacted. The OSHA sets and enforces protective workplace safety and health standards. The aim of this cross-sectional study was to assess the noise exposure in an endoscopic preparation room to investigate the relative risk for occupational health and to explore the balance between patient safety and labor health.

## 2. Methods

Under the aims and scope of the Kaohsiung Chang Gung Memorial Hospital occupational health and safety policy (OHSAS 18001), noise evaluations were performed to measure the noise levels in an endoscopic preparation room once a month from January to December 2013. The endoscopic preparation room was an enclosed area of around 3 × 3 square meters with a 10–15 Pa negative pressure ventilation system, 24°C room temperature, and 65% humidity, with 7 AERs including 5 for upper GI scopes and 2 for lower GI scopes. There are a total of 26 upper GI scopes in our endoscopic unit including 2 nasogastroscopes and 2 small intestinal scopes and 13 lower GI scopes including 1 sigmoidoscope and 4 endoscopic retrograde cholangiopancreatic scopes.

One endoscopic preparation nurse with 7 years of experience was responsible for the entire endoscopic manual washing, disinfecting, drying, and clinical preparation processes with an 8-hour TWA from Monday to Friday. The nurse reported to work from 8:00 a.m. to 5:00 p.m. including an hour-long lunch break. The main work activities of the nurse were collecting all of the used GI endoscopes, checking for water leakage, manually brushing the working channel of the scopes, and then placing them in the AERs. After HLD, a HPAJ was used to remove the residual water on the surface of GI endoscopes one by one. At the same time, 7 AERs were working, and a noise dosimeter was used to record the noise exposure of the nurse. As mentioned, the nurse came in at 8:00 a.m. and started preparing the GI endoscopes, the first of which required drying with the HPAJ after HLD with AER at about 9:00 a.m. This process took approximately 30 seconds to 1 minute, and the whole preparation process continued until 5:00 p.m. In total, 22842 HLD procedures were carried out in the year of the study period, with an average of 62.6 endoscopic procedures per day. Noise spectral data were measured during daily practice at the endoscopic preparation unit. The noise dosimeter was set following the OSHA criteria and then attached to the nurse's collar to record personal noise exposure during the working periods. The noise measurements in this study were performed to ascertain noise levels in terms of an 8-hour TWA from 9:00 a.m. to 5:00 p.m. during each working period in the endoscopic preparation room using the noise dosimeter.

### 2.1. Noise Measurements

**Figure 1:**
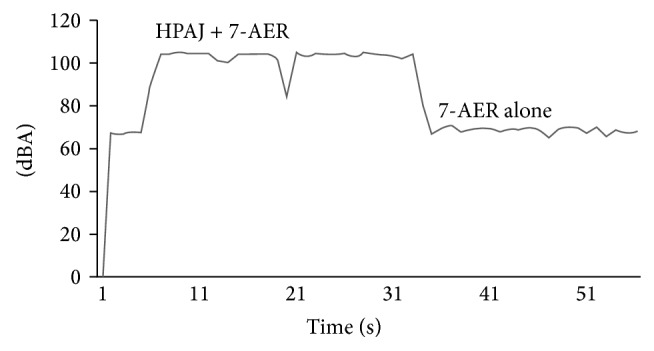
Noise measurements: the study group was the 7 AERs working combined with HPAJ (HPAJ + 7-AER); the control group was the 7 AERs working alone (7-AER).

Noise exposure in the endoscopic preparation room was assessed using a noise dosimeter, and the spectral analysis results were taken and divided into two groups: the study group, which included all of the 7 AERs working together combined with the HPAJ working at the same time, and the control group, which included the noise coming from the 7 AERs only (Figure 1).

The noise dosimeter (TDJ 814, TONDAJ instrument Co., Bao-An Shenzhen, China) was a sound level data logging meter with a USB attachment and computer software and followed the OSHA criteria including an exchange rate of 5 decibels. The frequency weighting was A, the response was slow, the criteria level was 90 dBA, and the threshold was 80 dBA. The 80 dBA threshold was used to measure the noise that the nurse identified during a walk around which may exceed 85 dBA on a TWA. The noise dosimeter readout was in percent noise dose exposure and the equivalent continuous A-weighted sound level in decibels for each minute during the period sampled. The noise level was presented in decibel A-scale (dBA) which refers to a human hearing threshold and was calculated for an 8-hour TWA for each period of work, while impulsive noise levels were presented in decibel C-scale (dBC) [7].

A windscreen provided by the dosimeter manufacturer was placed over the microphone during recordings. The information stored in the dosimeters was downloaded to a personal computer for interpretation with Windows computer software. The dosimeter was calibrated before and after the measurement periods according to the manufacturer's instructions.

### 2.2. Calculation Assessment

Analytical procedures of the United States Institute for Occupational Safety and Health (NIOSH) and the International Organization for Standardization (ISO) of noise-induced hearing loss were estimated to develop analytic models as the base of the present study.

#### 2.2.1. NIOSH Analytical Method

This was developed in 1972; in this method, it was created by a noise and health survey data, in which a total of 1172 men case data were collected and used with logistic regression of statistics to describe the correlation between hearing loss and its impact factors.

#### 2.2.2. ISO Method

In this method, baseline control teams are divided into two groups, one for ISO7029 method for rigorous screening persons with normal hearing otology; the other group is benchmarking, which has been recommended for all elected from among the towns of occupational noise exposure. On the application, if it involves issues such as compensation, more suitable for the former as a reference, but the latter was not filtered, groups' benchmarks may be more suitable as a general base of community workers noise exposure assessment study. In addition, the labor hearing threshold values were also added as a control group of another baseline.

In addition to the references from the original of databases of these two methods, we used software with logistic regression from the Institute of Occupational Safety and Health of Taiwan (http://www.iosh.gov.tw/default.aspx) for the hearing threshold values of the survey data, which were based on environment noise sound levels, exposure time, and age for correlation with the risk of hearing loss. This software was based on hearing protection plans and used CAD (Computer Aided Design) links to the database with Delphi 4.0 software from the Institute of Labor, OSHA, Ministry of Labor of Taiwan (http://www.ilosh.gov.tw/wSite/ct?xItem=3135&ctNode=282&mp=11). Data entered into the software included factory field name and basic data such as hearing check records to education training courses, noise environment determination, understanding factory noise distribution situation, investigating labor health situation, and protection by using case and problem.

For the software, the age of the worker needed to be more than 30 years and the exposed noise needed to be more than 90 dBA (Table 1). Our endoscopic preparation nurse was exposed to the HPAJ for 2 years from 31 years of age. Extension and design of pretendage of the labor were from 33 to 49 years of age with a fixed exposure period of 2 years to get to know the risk of the hearing loss >2.5 dB.

### 2.3. Statistical Analysis

The odds ratio was used with Statistics notes by Bland and Altman [8] and Hutchon [9]. Comparisons of parameters for the risk of hearing loss between the combined HPAJ + 7-AER and the 7-AER alone groups were performed using the *X* ^2^ test, Fisher's exact test, and Student's *t*-test with SPSS software (version 12.0; SPSS, Chicago, IL, USA). *P* values less than 0.05 were considered to be statistically significant.

### 2.4. Ethics Statement

All clinical investigations were conducted according to the principles expressed in the Declaration of Helsinki. Written informed consent was obtained from all participants for their information to be stored in the hospital database and used for research. This study was approved by the Institutional Review Board of Chang Gung Memorial Hospital Ethics Committee (number 102-3890B).

## 3. Results

**Figure 2:**
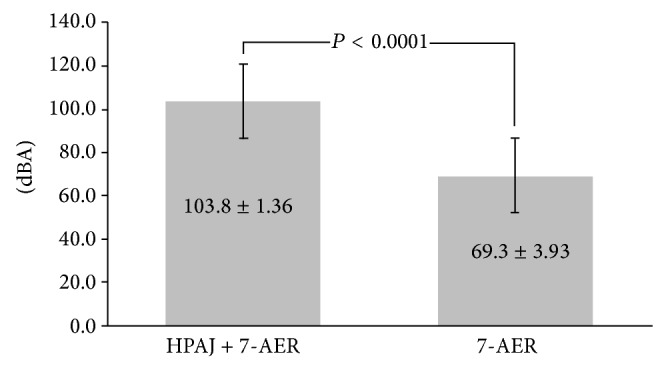
The noise level of the HPAJ and 7-AER group was significantly higher than that of the 7-AER alone group (108.3 ± 1.36 versus 69.3 ± 3.93 dBA, *P* < 0.0001).

The peak of the noise spectrum of the combined HPAJ and 7-AER group was significantly higher than that of the 7-AER alone group (108.3 ± 1.36 versus 69.3 ± 3.93 dBA, *P* < 0.0001) (Figure 2). Depending on the different noise exposure, the risk of the hearing loss (HL > 2.5 dB) with 2-year exposure period was 0.1% at 90 dBA, 0.5% at 95 dBA, and 1.4% at 100 dBA. We tried to pretend that the exposure period extended to 7 years and then calculated the risk of hearing loss (HL > 2.5 dB) which was increased up to 2.15% at 90 dBA, 11.6% at 95 dBA, and 51.3% at 100 dBA, respectively (Table 2). The odds ratio was 49.1 with the 95% confidence interval ranging from 11.9 to 203.6 with the program written by Hutchon [9].

## 4. Discussion

Kaohsiung Chang Gung Memorial Hospital is a medical center with 2,715 beds and over 6,900 outpatients and 370 emergency patients. We have a policy of a safe and healthy environment in this high-load teaching hospital and have established and implemented occupational health and safety for the promotion, prevention, and protection of the physical and mental health of all parties. In addition, we promote a safe occupational health concept and maintain and continually improve our occupational health and safety policy with the target of being accident-free. This prospective, cross-sectional, and regional study was mainly performed to assess noise exposure inside our endoscopic preparation room. The maximum noise exposure when both the 7 AERs were processing and the HPAJ was being used was significantly greater than for the 7-AER only (103.8 ± 1.36 versus 69.3 ± 3.93 dBA, *P* < 0.0001), and it was much higher than the permissible noise exposure of 85 dBA [10]. According to the permissible occupational noise exposure 1910.95 of the OSHA [11], an 8-hour TWA above 90 dBA is not permitted, and the noise exposure for 1.0 to 1.5 hours should have an upper limit between 102 and 105 dBA (Table 3) [11]. We also applied the calculation program interface from the OHSA for the prediction of hearing loss (>2.5 dB) (http://www.ilosh.gov.tw/wSite/ct?xItem=3135&ctNode=282&mp=11) and found that even a young person as in our case had a 1.4% risk of hearing loss (>2.5 dB) with 2-year exposure to a 100 dBA noise level (Tables 1 and 2). When we tried to extend the accumulated exposure period to 7 years, the program indicated risks of 2.15%, 11.6%, and 51.3% hearing loss (>2.5 dB) with 90 dBA, 95 dBA, and 100 dBA noise levels, respectively (Table 2). HPAJ was thus indicated to be unsuitable for indoor use and was withdrawn from our endoscopic preparation room immediately. Our endoscopic preparation nurse underwent hearing tests and fortunately no defects were noted.

Therefore, the use of an HPAJ to increase the reuse rate of GI endoscopes proved to be a wrong policy. For labor protection, all exposed workers must be provided with, and must use, personal protective equipment to reduce the sound to permissible levels of exposure. In addition, employees who are exposed to an 8-hour TWA noise level of 85 dBA or greater and who have experienced a standard threshold shift must be provided with, and must use, hearing protectors with sufficient attenuation to reduce noise levels below 85 dBA [11]. Some working environments such as medical services [7], schools [12], or restaurants [13] are not suitable for ear protection and differ from outdoor working environments [14]. In the case of our unit, the main factor is the high load of GI endoscopies requested in a large academic hospital. Purchasing more GI endoscopes to increase the number directly or indirectly by employing two or three endoscopic preparation nurses to separate the 8-hour TWA is viable option; however, this will incur a heavy burden on the hospital (Table 4). After we stopped using the HPAJ, we consulted with infection experts who suggested that manual wiping with aseptic cloths should be acceptable. Other than that, we have also implemented a monitoring program with two investigations per year and made it easier for nurses to lodge complaints. For the working nurses, we also supply ear protection devices, regularly monitor hearing with standard hearing tests every year, ensure that all the working places do not exceed an 8-hour TWA of 85 dBA, and implemented strategies for compulsory vacation (Table 4). Noise can not only induce hearing loss but also result in other physiological and psychological effects [15, 16].

In conclusion, working in an endoscopic preparation room, the noise generated by using a HPAJ over an 8-hour TWA seriously affects the occupational health and violates safety management systems. Patient safety and workers health are both important in an academic hospital.

## Figures and Tables

**Table 1:** The risk of hearing loss (>2.5 dB) was increased depending on the exposure period and age of the endoscopic preparation nurse compared with the basic sound level 80 dBA in the endoscopic preparation room.

Compared with basic sound level		80 dBA		

**Exposure noise**		**90 dBA**	**95 dBA**	**100 dBA**

Exposure period^*^	Exposure age^*^	Risk of the hearing loss (HL > 2.5 dB)		

2 years	31 y/o	0.1%	0.5%	1.4%

Fixed period	Pretend labors			

2 years	33 y/o	0.1%	0.6%	1.6%
2 years	35 y/o	0.2%	0.7%	1.8%
2 years	37 y/o	0.2%	0.8%	2.1%
2 years	39 y/o	0.2%	0.8%	2.4%
2 years	41 y/o	0.2%	1.0%	2.6%
2 years	43 y/o	0.3%	1.1%	2.9%
2 years	45 y/o	0.3%	1.2%	3.2%
2 years	47 y/o	0.3%	1.3%	3.5%
2 years	49 y/o	0.3%	1.4%	3.8%

^*^According to the interface program from the Institute of Occupational Safety and Health of Taiwan (http://www.iosh.gov.tw/default.aspx); the exposure period needed >2 years and worker's age >30 years at least.

**Table 2:** The expected risk of hearing loss (>2.5 dB) was significantly higher after 7 years than in 2 years of the noise exposure period for the endoscopic preparation nurse.

Age	31 years old		
Working experience	7 years		
Exposure years	2 years		
Basic sound level	80 dBA		

Exposed noise	90 dBA	95 dBA	100 dBA

Hearing loss	>2.5 dB		

Accumulated exposure period			
If 2 years	0.1%	0.5%	1.4%
If 7 years	2.15%	11.6%	51.3%

The odds ratio was 49.1 with 95% confidence interval from 11.9 to 203.6 (http://www.hutchon.net/ConfidORselect.htm).

**Table 3:** Permissible noise exposures by the Occupational Safety and Health Administration (OSHA)^*^.

Duration per day (hours)	Sound level dBA slow response
8	90
6	92
4	95
3	97
2	100
$1\frac{1}{2}$	102
1	105
$\frac{1}{2}$	110
$\frac{1}{4}$ or less	115

^*^ https://www.osha.gov/pls/oshaweb/owadisp.show_document?

p_table=STANDARDS&p_id=9735#1910.95(b)(1).

**Table 4:** Noise exposure prevention in an endoscopic preparation room.

For the unit	For the nurse
^■^Stop using a HPAJ	^■^Ear protection device
^■^Manually wipe with aseptic cloths	^■^Regular hearing monitoring
^■^Implement a monitoring program	^■^Not exceeding an 8-hour TWA of 85 dBA
^■^Implement a complaints policy	^■^Strategy for compulsory vacation
^□^Increase endoscopies	^□^Increasing to 2~3 endoscopic nurses

^■^Available; ^□^not available.

## Abbreviations

AER: Automatic endoscopic reprocessor
dB: Decibels
dBA: Decibels, A-weighted scale
HL: Hearing loss
HPAJ: High pressure air jet
ISO: International Organization for Standardization
NIOSH: National Institute for Occupational Safety and Health of the United States
OSHA: Occupational Safety and Health Administration
TWA: Time-weighted average.
